# Epidemiology of Food Choking Deaths in Japan: Time Trends and Regional Variations

**DOI:** 10.2188/jea.JE20200057

**Published:** 2021-05-05

**Authors:** Yuta Taniguchi, Masao Iwagami, Nobuo Sakata, Taeko Watanabe, Kazuhiro Abe, Nanako Tamiya

**Affiliations:** 1 Graduate School of Comprehensive Human Sciences, University of Tsukuba, Ibaraki, Japan; 2 Department of Health Services Research, Faculty of Medicine, University of Tsukuba, Ibaraki, Japan; 3 Health Services Research and Development Center, University of Tsukuba, Ibaraki, Japan; 4 Department of Public Health, Graduate School of Medicine, The University of Tokyo, Tokyo, Japan

**Keywords:** food suffocation, older people, airway obstruction, epidemiology, Japan

## Abstract

**Background:**

With increasing age globally, more people may become vulnerable to food choking. We investigated the nationwide epidemiology of food choking deaths in Japan.

**Methods:**

Using Japanese Vital Statistics death data between 2006 and 2016, we identified food choking deaths based on the 10th revision of the International Statistical Classification of Diseases code W79 (Inhalation and ingestion of food causing obstruction of respiratory tract) as a primary diagnosis. We assessed the demographics of people with food choking deaths; temporal trends of food choking deaths by the year (overall and by age group), the day of year; and prefecture variations.

**Results:**

Overall, 52,366 people experienced food choking deaths (median age, 82 years, 53% were male, and 57% occurred at home). The highest numbers occurred January 1–3, and were lowest in June. Despite a stable total number of cases at around 4,000 yearly, from 2006 to 2016 the incidence proportion declined from 16.2 to 12.1 per 100,000 population among people aged 75–84 years. Among people ≥85 years, the incidence proportion peaked at 53.5 in 2008 and decreased to 43.6 in 2016. The number of food choking deaths varied by prefecture.

**Conclusions:**

There are temporal and regional variations of food choking deaths in Japan, possibly due to the consumption of Japanese rice cake (mochi), particularly over the New Year’s holiday.

## INTRODUCTION

The swallowing function of people deteriorates with age and older people are more likely to choke on food.^1^^–^^3^ With increasing numbers and proportions of older people globally,^4^ more people may become vulnerable to food choking.

Japan has the highest proportion of older people worldwide,^4^ and approximately 4,600 people died from food suffocation in 2018.^5^ Only a few studies have identified the characteristics of Japanese patients with choking incidents.^6^^–^^8^ Two studies based in single facilities reported higher incidences of choking on food among older people,^6^^,^^7^ particularly in January.^7^ A study based in one prefecture showed a higher incidence of out-of-hospital cardiac arrest specifically due to choking on Japanese rice cake (mochi) during the first 3 days of the New Year.^8^

However, due to their narrow demographic and regional scope, these results are of limited generalizability.^6^^–^^8^ Because eating habits vary across Japan, it is warranted to investigate national and prefectural variations in food choking deaths. In addition, with a recent focus by the authorities on food suffocation,^9^^,^^10^ it is of interest whether food choking deaths (overall and by age group) are increasing or decreasing. Finally, information on temporal trends of food choking deaths will be useful for public awareness campaigns focusing on prevention.

The present study uses mortality data to describe characteristics of food choking deaths across the country, with attention to age and temporal and regional variations.

## METHODS

### Data sources and definition of food choking deaths

We obtained Japan Vital Statistics mortality data for 2006 to 2016 from the Ministry of Health, Labour and Welfare under Statistics Act, Article 33. The study was approved by the Ethics Committee of the University of Tsukuba (approval no. 1324-2). Individual participants’ consent was waived because of the anonymized nature of the data.

The Vital Statistics System of Japan records every death, including the date, place, and cause of death.^11^ In addition, for deaths due to external causes the place of occurrence (home, residential institution, school, public administrative area [eg, school and hospital], trade and service area [eg, restaurant], or other places) is recorded. We identified individuals with external cause of death code W79 (Inhalation and ingestion of food causing obstruction of respiratory tract), according to the 10th revision of the International Statistical Classification of Diseases (ICD-10).

### Statistical analysis

The mortality data provided basic demographic characteristics of people with food choking deaths, specifically, sex, age at death, place of occurrence (home, residential institution, public administrative area [eg, school and hospital], trade and service area [eg, restaurant], or other places), and date of death. We excluded anyone with missing data on one or more of these variables. Age was categorized as <75, 75–84, and ≥85 years. We calculated the incidence proportion as the number of food choking deaths divided by the number of people living in Japan as reported by the Population Estimates^12^ for each year between 2006 and 2016, and by age group. We also calculated month-specific numbers of food choking deaths, using the 2006 to 2016 data combined. Lastly, we evaluated regional variations in the number of food choking deaths among the 47 prefectures by calculating standardized mortality ratios (SMR). We calculated the SMR by dividing the average annual number of food choking deaths in each prefecture from 2006 to 2016 by the total expected deaths in that prefecture, using the five-year age structure of Japan as reference.^12^

We carried out the analyses using Stata 15 (StataCorp, College Station, TX, USA) and Microsoft Excel 16.33 (Microsoft, Redmond, WA, USA).

## RESULTS

**Figure 1.**
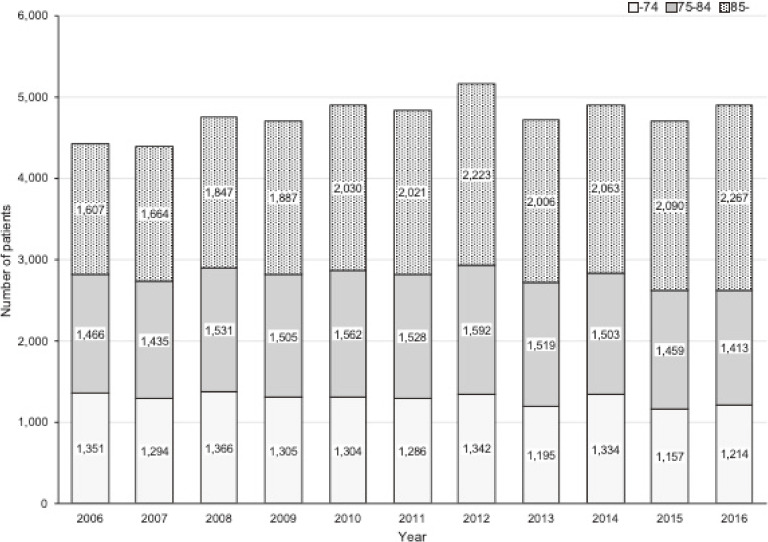
Annual trends in the number of food choking deaths in Japan between 2006 and 2016, overall and by age group

**Figure 2.**
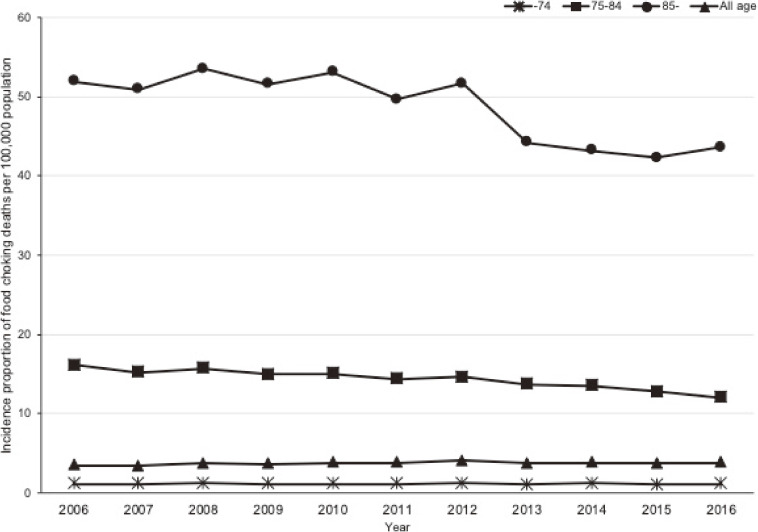
Annual trends in the incidence proportion of food choking deaths in Japan between 2006 and 2016, overall and by age group

A total of 13,407,914 people died between January 1, 2006 and December 31, 2016 in Japan. After excluding six people with missing values, we identified 52,366 people with food choking deaths; 53% (27,881/52,366) were male, and the median age of those included in this analysis was 82 (inter-quartile range, 74–89) years; 73.0% (38,218/52,366) were 75 years or older. By place of occurrence, 56.9% (29,777/52,366) of food choking occurred at home, 18.1% (9,488/52,366) at residential institution, 9.8% (5,150/52,366) at public administrative area (eg, school and hospital), and 2.9% (1,533/52,366) at trade and service area (eg, restaurant). The percentage of home-based food choking increased with age, with 8.4% (1,183/14,148), 15.7% (2,595/16,513), and 26.3% (5,710/21,705) occurring among people aged <75, 75–84, and ≥85 years, respectively. In contrast, the proportions occurring in public administrative areas and in trade and service areas decreased in older age groups. Overall, the number of food choking deaths was relatively stable at approximately 4,000 each year (Figure 1). However, the incidence proportion for people aged 75–84 years declined steadily, from 16.2 per 100,000 population in 2006 to 12.1 per 100,000 population in 2016 (Figure 2). Among people aged ≥85 years, the incidence proportion peaked at 53.5 per 100,000 population in 2008 and decreased to 43.6 per 100,000 population in 2016.

**Figure 3.**
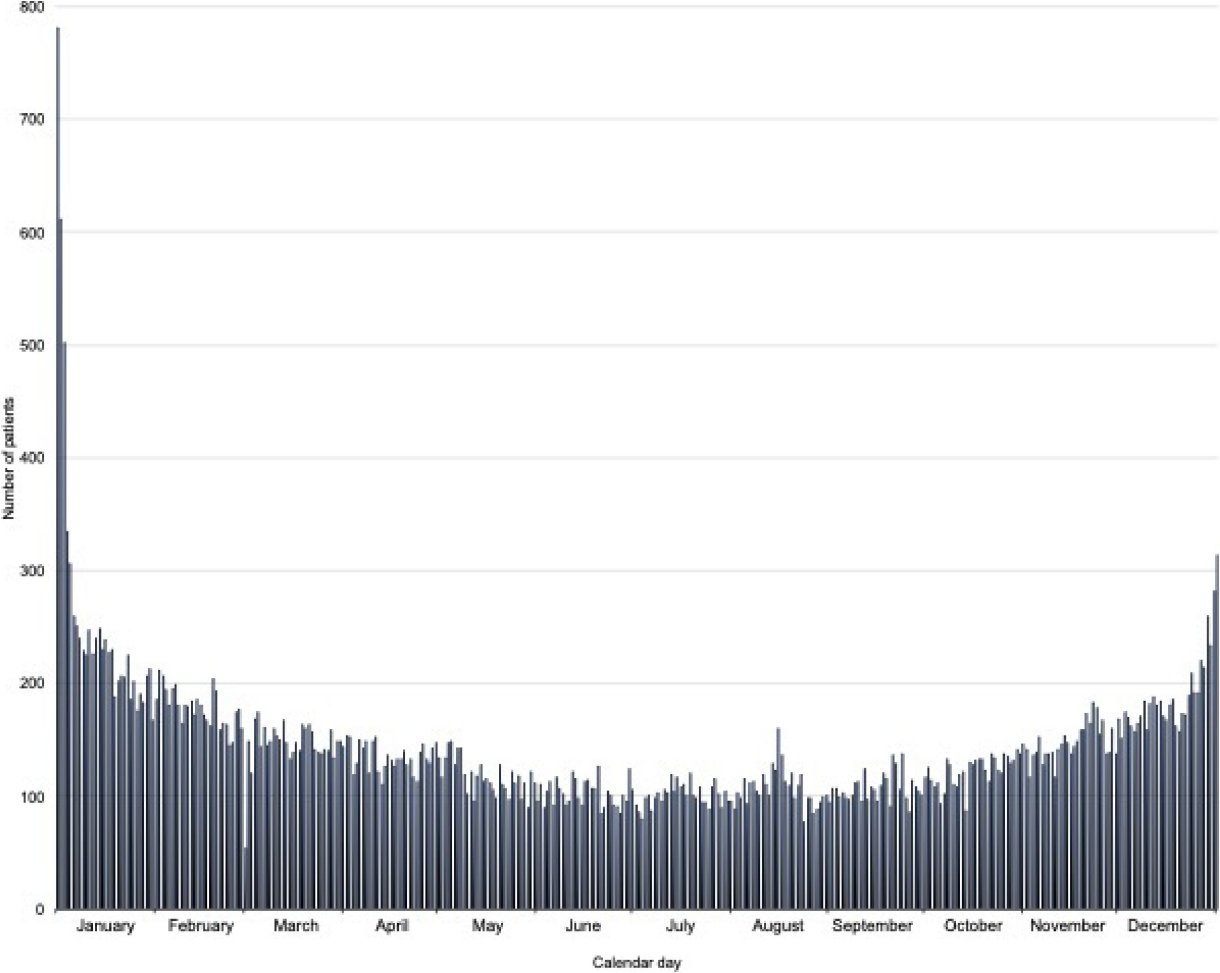
Temporal trend in the monthly number of food choking deaths in Japan (2006 through 2016 data combined)

**Figure 4.**
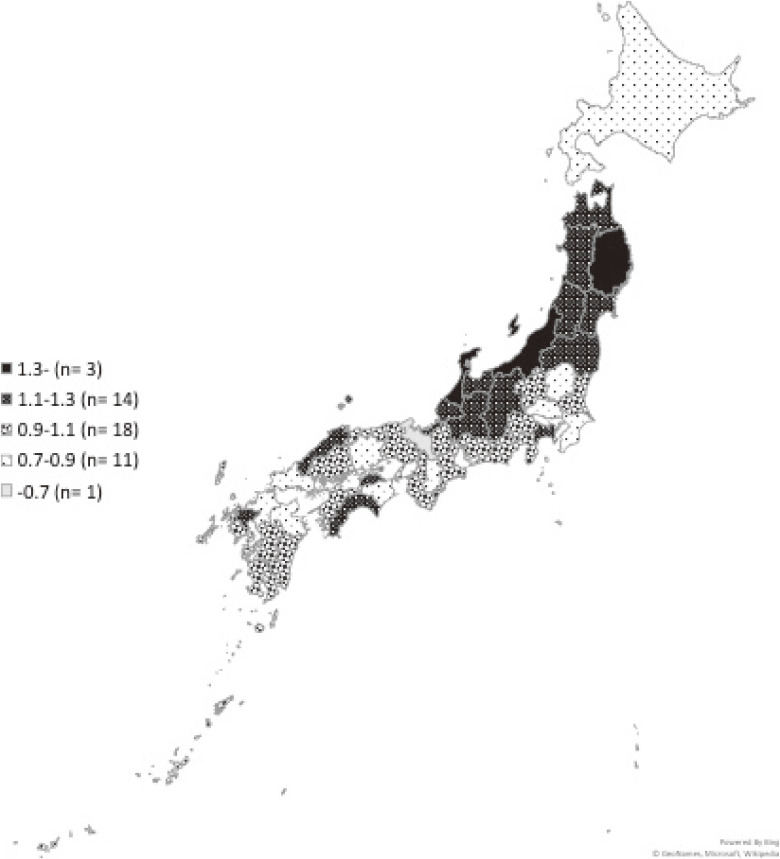
Geographic variations in the incidence proportion of food choking deaths. Note: We calculated the standardized mortality ratios (SMR) by dividing the average annual number of food choking deaths in each prefecture from 2006 to 2016 by the total expected deaths using the age distribution of the population of Japan in 2011.

Over the total period, food choking deaths were highest in January and lowest in June (Figure 3). Further, deaths were highest on January 1, followed by January 2 and January 3, with 782, 611, and 502 deaths, respectively. The SMR varied among prefectures: highest in Niigata (1.38) and lowest in Kyoto (0.60) (Figure 4).

## DISCUSSION

To our knowledge, this study is the first to demonstrate demographics, time trends, and regional variations in food choking deaths in Japan nationally. The results showed that the number of deaths due to food suffocation was highest on New Year’s Day, frequently occurred at home, and was highest among people aged over 75 years. We also found regional variations in food choking deaths, which were highest in Niigata Prefecture and lowest in Kyoto Prefecture.

Previous Japanese studies in specific hospitals^6^^,^^7^ and a single prefecture^8^ also reported more frequent food choking deaths among older people, at home, and in January. Our national-level analyses were compatible with these previous results but revealed greater detail.

First, we showed that the number of food choking deaths was highest in January, specifically on January 1. “Zoni”, a soup containing Japanese rice cake (mochi), is a traditional New Year’s Day meal in Japan.^13^ Japanese rice cake is known as a major cause of food choking in Japan because of its soft and sticky nature.^14^ In Osaka Prefecture, it was reported that 24.5% of out-of-hospital cardiac arrests from choking on rice cake occurred during the first 3 days of the New Year.^8^ A high incidence of choking among older people is consistent with results of studies conducted in the United States,^15^^,^^16^ the United Kingdom,^17^ Australia,^18^ and Taiwan.^19^ However, seasonal variations, such as Japan’s high frequency of deaths in January, were not observed in the United Kingdom study,^17^ possibly attributable to the Japanese tradition of eating rice cake to celebrate the New Year.

Second, as this study showed, despite Japan’s stable number of food choking deaths, the incidence among people over 75 years old is decreasing. Entities such as the Consumer Affairs Agency,^9^ the Tokyo Fire Department,^10^ and mass media^20^ are delivering warnings about the risk of choking on food, and on rice cake in particular. We speculate that the public has become more aware of the choking risk, although we are unable to estimate the impact of public campaigns. Further, the declining consumption of rice cake over the past decade, from 2,872 grams per household per year (2006) to 2,459 grams (2016) may have resulted in fewer choking deaths.^21^

Third, we found that the incidence proportion of food choking deaths varied among prefectures. We propose two possible mechanisms for these variations. First, the prefecture with the highest incidence of food choking deaths (Niigata) is also known for high Japanese rice cake consumption.^21^ Of 47 prefectural capital cities, Niigata City consumed the eighth highest level between 2010 and 2012. Second, differences in the emergency medical system (EMS) between prefectures may affect the probability of death for people who experience food choking. Higher mortality rates are expected in prefectures that have longer times between EMS notification and first treatment at hospital. Indeed, in 2011, the prefecture with the highest SMR (Niigata) was 41st (out of 47) in average time from emergency call to arrival at the hospital (40.5 minutes).^22^ At the same time, however, the prefecture with the 3rd highest SMR (Ishikawa) had the 7th shortest time from emergency call to hospital arrival (31.0 minutes), and so the relationship is not consistent. Regional variation in food choking deaths is likely multifactorial, which requires further investigation.

Our study has several limitations. First, the Vital Statistics database does not include information on the type of food causing suffocation. Previous studies reported Japanese rice cake as the most common cause of food choking,^14^ and we assume that it might have played a major role in this study but are unable to examine the extent to individual types of food influenced the number of choking deaths. From a previous study, Japanese rice cake accounted for 24.5% of the food chokings,^14^ and the survival outcome after out-of-hospital cardiac arrests was reported to be worse for other food types compared to rice cake.^8^ A more detailed study is needed about suffocation from different food types (eg, candy, meat), and cause-specific solutions to food choking deaths.

There may also have been a time lag between the occurrence of food choking and death in cases where patients were resuscitated. Therefore, the reported incidence of food choking on New Year’s Day may be an undercount when based on the day of death. Finally, the Vital Statistics database lacked detailed information on individual-level factors, such as underlying medical conditions or socio-economic factors that influence the occurrence of food choking. For example, people with lower educational attainment may be more vulnerable to food choking because of poor oral health (eg, number of teeth), or less influenced by warnings about food choking. However, we could not investigate the association between these factors and food choking deaths.

In conclusion, approximately 4,000 food choking deaths occurred in Japan yearly from 2006 to 2016; over that time period the incidence among people over 75 years old decreased. The number of food choking deaths was higher among older people, in January (particularly on New Year’s Day), and at home. Regional variations existed between prefectures, and these may be related to the amount of Japanese rice cake consumed. To reduce the number of food choking deaths a focus on older people, particularly during the New Year holiday, may be effective.
